# Mechanosensitive Ion Channel PIEZO1 Signaling in the Hall-Marks of Cancer: Structure and Functions

**DOI:** 10.3390/cancers14194955

**Published:** 2022-10-10

**Authors:** Fuqiang Zhao, Lei Zhang, Mankun Wei, Wei Duan, Shourong Wu, Vivi Kasim

**Affiliations:** 1 Key Laboratory of Biorheological Science and Technology, Ministry of Education, College of Bioengineering, Chongqing University, Chongqing 400044, China; 2 The 111 Project Laboratory of Biomechanics and Tissue Repair, College of Bioengineering, Chongqing University, Chongqing 400044, China; 3 Chongqing Key Laboratory of Translational Research for Cancer Metastasis and Individualized Treatment, Chongqing University Cancer Hospital, Chongqing University, Chongqing 400030, China

**Keywords:** Piezo1, ion channel, mechanical signals, hallmarks of cancer, tumorigenesis

## Abstract

**Simple Summary:**

Tumor cells obtain various unique characteristics, which known as hallmarks of cancers, including sustained proliferative signaling, apoptosis resistance, and metastasis. These characteristics are crucial for tumor cells survival and for supporting their rapid growth. Studies have revealed that tumorigenesis is also accompanied by alteration in mechanical properties. Tumor cells could sense various mechanical forces, such as compressive force, shear stress, and portal vein pressure, which in turn could affect tumor progression. Piezo1 is a mechanically sensitive ion channel protein that can be activated mechanically, and is closely related to various diseases. Recent studies showed that Piezo1 is overexpressed in numerous tumors and is associated with poor prognosis. Furthermore, previous studies revealed that Piezo1 mediates these cancer hallmarks, and thus links up mechanical forces with tumor progression. Therefore, the discovery of Piezo1 provides a new insight for elucidating the mechanism of tumor progression under a mechanical microenvironment.

**Abstract:**

Tumor cells alter their characteristics and behaviors during tumorigenesis. These characteristics, known as hallmarks of cancer, are crucial for supporting their rapid growth, need for energy, and adaptation to tumor microenvironment. Tumorigenesis is also accompanied by alteration in mechanical properties. Cells in tumor tissue sense mechanical signals from the tumor microenvironment, which consequently drive the acquisition of hallmarks of cancer, including sustained proliferative signaling, evading growth suppressors, apoptosis resistance, sustained angiogenesis, metastasis, and immune evasion. Piezo-type mechanosensitive ion channel component 1 (Piezo1) is a mechanically sensitive ion channel protein that can be activated mechanically and is closely related to various diseases. Recent studies showed that Piezo1 mediates tumor development through multiple mechanisms, and its overexpression is associated with poor prognosis. Therefore, the discovery of Piezo1, which links-up physical factors with biological properties, provides a new insight for elucidating the mechanism of tumor progression under a mechanical microenvironment, and suggests its potential application as a tumor marker and therapeutic target. In this review, we summarize current knowledge regarding the role of Piezo1 in regulating cancer hallmarks and the underlying molecular mechanisms. Furthermore, we discuss the potential of Piezo1 as an antitumor therapeutic target and the limitations that need to be overcome.

## 1. Introduction

Mechanical forces are pervasive in living organisms [1]. For decades, researchers have focused on the mechanical microenvironments to which various cells are subjected, providing more evidence about the links between physical conditions and biological pathways [2]. Several types of mechanical forces have been reported to play crucial roles in physiological conditions [3]. For example, endothelial cells in the blood and lymphatic vessels constantly interact with luminal and abluminal extracellular environments that confer endothelial physical forces, including shear stress, stretching, and stiffness [4]. Cells could sense these mechanical forces and convert them into biochemical signals through a mechanical transducer-mediated process, which ultimately affects biological behaviors, such as cell proliferation, survival, and differentiation [5]. Furthermore, abnormal remodeling of the extracellular mechanical environment and/or defects in the correct response to mechanical cues lead to various diseases, such as fibrosis, muscle disease, and cancer [6]. Therefore, mechanical cues play important roles in physiological and pathological processes [7], and more systematic, detailed insights into the key mechanical sensors and mechanisms involved in mechanical transduction are essential for developing new therapeutic strategies against mechanical force-related diseases [8].

Piezo-type mechanosensitive ion channel component 1 (Piezo1), originally called Fam38A, is a core component of the mechanically activated ion channel [9,10,11]. It has attracted extensive attention since it was identified as a mechanically sensitive protein by Ardem Patapoutian, who won the Nobel Prize in Physiology in 2021 for this discovery [12]. Studies have demonstrated that Piezo1 is a multipass transmembrane protein highly conserved in vertebrates, with homologs in invertebrates, protozoa, and plants [13,14]. Piezo1 has special ion channel properties, as it could allow non-selective cation penetration once the cell senses a mechanical stimulus, allowing potassium, sodium, magnesium, and calcium (Ca^2+^) ions to pass through the cell membrane, with a slight preference for Ca^2+^ [15,16,17]. Piezo1 senses mechanical cues, including membrane stretch [18], shear flow [19], substrate stiffness [20], and tissue compression [21]; thereby causing the influx of Ca^2+^, which in turn acts as the second messenger of signal transduction and controls various physiological processes [22]. Hence, Piezo1 links mechanical forces to biological signals, and mediates these mechanical responses as a type of biological mechanotransducer to drive physiological and pathological processes, such as bone homeostasis, inflammation, and atherosclerosis [23].

Recently, Piezo1 has been shown to participate in mediating multiple cancers (Table 1) [24,25,26,27,28,29,30,31,32,33,34,35,36,37,38,39,40,41,42,43,44,45]. Tumorigenesis is an intricate process involving dynamic changes in the genome that drive malignancy transformation [46,47]. These transformed cells exhibit unique traits, such as sustained proliferative signaling, evading growth suppressors, and apoptosis resistance, which are known as the hallmarks of cancer [48]. Meanwhile, tumor tissues showed abnormal stiffness, compressive force, and shear stress compared with normal tissues [49]. These mechanical forces can be sensed by tumor cells, and, in turn, affect their characteristics, including proliferation, angiogenesis, and invasion potentials [50]. Furthermore, Piezo1 is highly expressed in various tumors and is associated with poor prognoses in most cancers (Table 1) [44,51]. As we discuss this further in the following sections and summarize it in Table 2, activation of the Piezo1 channel, which allows Ca^2+^ to pass through the cell membrane and in turn affects various downstream signaling pathways, is closely linked with multiple hallmarks of malignancies.

## 2. Structure of the PIEZO1 Ion Channel

Mammalian Piezo1 is an evolutionarily conserved ion channel protein containing more than 2500 amino acid residues with a molecular weight of about 300 kDa. Piezo1 forms an about 900 kDa homotrimer with a trimeric propeller-like structure containing a central cap and three distal blade-like structures as its extracellular domains, together with 42 transmembrane segments and three long beam-like structures that are located at the intracellular region [58]. Topology determination demonstrated that the cap structure is formed by residues ranging from 2214 to 2457, termed the C-terminal extracellular domain (CED), and is located at the extracellular side. Meanwhile, each blade structure, which is highly curved and mobile, and whose volume is comparable to the cap region, is composed of approximately 700 amino acid residues forming nine repetitive units, each of which contains four transmembrane helices [59].

**Figure 1:**
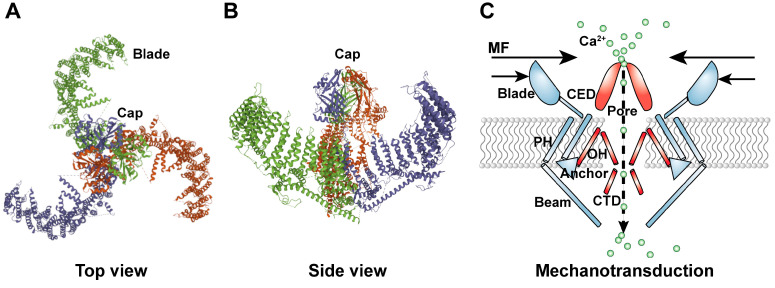
**Structure of Piezo1 Channel**. (**A**) Top view and (**B**) side view of the Piezo1 ion channel as reported in the Protein Data Bank database (http://www.rcsb.org/pdb/home/home.do, accessed on 10 September 2022, structure ID: 6LQI). The major elements composing the ion channel are shown. (**C**) Schematic diagram of the mechanotransduction and pore modules of the Piezo1 channel. CED: C-terminal extracellular domain; CTD: intracellular C-terminal domain; MF: mechanical forces; OH: outer helix; PH: peripheral helix.

The transmembrane region comprises transmembrane segments forming three peripheral wings and a central pore module [60]. The core transmembrane segments consist of the inner helix (IH), which is the last transmembrane segment at the C-terminus, and the outer helix (OH), which is likely the second-to-last transmembrane segment. Meanwhile, peripheral transmembrane arrays are referred to as the peripheral helix (PH). The 12 PHs of the same monomer are organized into six helical pairs and are connected to the extracellular blade. The OH structure is connected to PH through an anchor consisting of four continuous α-helices. The anchor exists at the interface of the two adjacent subunits, forming a unique hairpin structure parallel to the membrane surface. Furthermore, the intracellular C-terminal domain (CTD), which is formed by 2481 to 2547 amino acid residues, is an important section of the ion channel. The CTD interacts with the beam, whose diameter is approximately 90 nm. The beam originates from the lower end of the PH in the transmembrane region and ends near the central axis of the channel complex. The special architecture of the three beams suggests that not only can they support the transmembrane region but also transmit conformational changes from extracellular blades [60]. These modules, including three beams, three blades, a central cap, CTD, and transmembrane segments, collectively form the basic physical skeleton of the Piezo1 homotrimer and are crucial for its function as a mechanically sensitive ion channel (Figure 1). The Piezo1 channel has a delicate force-sensing and mechanotransduction mechanism. The CED, CTD, and transmembrane segments constitute the ion permeation pathway, determining the ion conduction and selectivity of the Piezo1 channel, and subsequently, the Piezo1’s pore properties [61]. The CED constituted the cap structure, which is responsible for selecting cations rather than anions to enter the pore and ensuring efficient ion conduction by electrostatics. The CTD formed an intracellular vestibule; by virtue of two acidic residues (E2,495 and E2,496), the CTD structure is crucial for determining ion selectivity between divalent and monovalent cations and unitary conductance [62]. The Piezo1 channel could also widen the ion-conducting pore, allowing organic cations, such as tetramethyl ammonium and tetraethyl ammonium, to permeate the channel [63]. The non-pore-containing region, which consists of residues 1 to 2190 at the N-terminal region, functions distinctly from the pore module. The non-pore module can confer mechanosensitivity to the mechano-insensitive trimeric acid-sensing ion channel-1, allowing it to function as an intrinsic mechanotransduction module that sufficiently gates its separate pore module [63]. Taken together, the Piezo1 channel employs mechanotransduction and ion-conducting modules to accomplish its specialized functions in mechanical forces sensing, gating, and ion conduction.

## 3. Roles of PIEZO1 in Cancer Hallmarks

**Figure 2:**
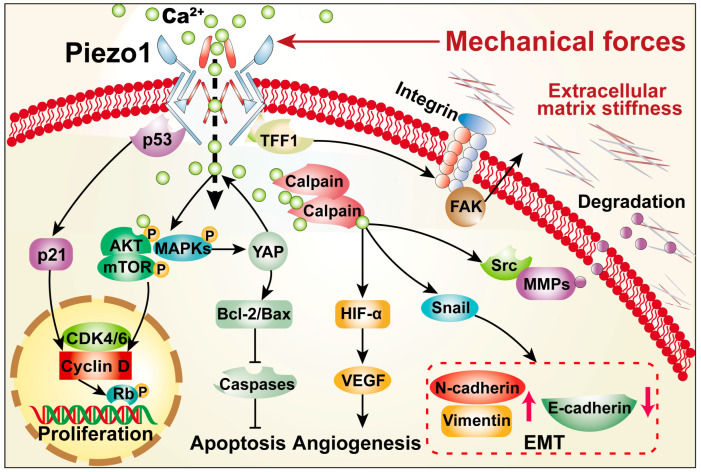
**Schematic Diagram of Piezo1 Regulation on Hallmarks of Cancer**. Mechanical stimulation triggers Piezo1 regulation on various hallmarks of cancer, especially sustained proliferative signaling, evading growth suppressors, apoptosis resistance, sustained angiogenesis, and metastasis.

Tumor progression is accompanied by a significant increase of solid tumor structural components, such as cancer cells, stromal cells, and extracellular matrix constituents, which lead to the increase of tumor stiffness, and thereby generate solid compression forces [64]. Solid compression forces in turn promote resistance to blood flow and interstitial fluid by compressing blood and lymphatic vessels, leading to the increase of fluid pressure and shear forces [65]. These mechanical forces in turn alter the properties of tumor cells and promote the cancer hallmarks, including evading growth suppressors, apoptosis resistance, sustained angiogenesis, and metastasis [66]. Furthermore, as will be summarized in the following sections, recent studies have shed light on the role of Piezo1 as a mechanical force sensor and transducer in various cancer hallmarks, linking up the mechanical sense with the biological properties of tumor cells (Figure 2) [67].

### 3.1. Sustained Proliferative Signaling

Limitless cell proliferation is one of the most obvious and important cancer hallmarks [48]. Normal cells need to be stimulated by pro-mitotic growth signals to exit from the quiescent state and enter the active proliferation state. However, external growth signals are not necessary for the proliferation of tumor cells [68]. Tumor cells can obtain sustained proliferation signals due to the widespread presence of dominant oncogenes in tumor cells and the overactivation of growth signaling pathways [69]. Previous studies have shown that tumor cells could obtain sustained proliferative signals by sensing mechanical signals [70]. Compared with the corresponding para-carcinoma tissues, Piezo1 is highly expressed in clinical hepatic carcinoma, breast cancer, and pancreatic cancer tissues [33]. Furthermore, its expression exhibits strong positive correlations with tumor markers, such as epidermal growth factor receptor (EGFR) and p38 mitogen-activated protein kinases (MAPK) [33]. Previous studies revealed that increasing Piezo1 expression greatly enhanced the proliferation potentials of various tumor cell lines, including malignant melanoma, oral squamous cell carcinoma, and hepatocellular carcinoma (HCC), by affecting the protein kinase B (AKT)/mechanistic target of rapamycin (mTOR), MAPK, and transforming growth factor-β (TGF-β) pathways, respectively [37,40,53]. Meanwhile, suppressing its expression canceled its oncogenic effect, significantly reducing the size and weight of the tumors formed in the xenograft model [31,54].

Abundant evidence shows that Piezo1 regulates the expression of crucial genes in the growth signaling pathway to control proliferation. MAPK is a major component of numerous signal transduction cascades that transmit upstream signals to downstream response molecules by sequential phosphorylation [71]. MAPK cascade could transmit growth signals from the cell surface to the nucleus; as a result, it is crucial for maintaining cell survival and proliferation potential [32]. Piezo1 promotes MAPKs phosphorylation and, subsequently, HCC proliferation by increasing the Ca^2+^ influx. The yes-associated protein (YAP) signaling cascade, which is regulated by Piezo1 during the differentiation of human neural stem cells and the development of zebrafish hearts, is also upregulated by Piezo1/MAPK signaling pathway, thereby promoting tumor cell proliferation [52]. Intriguingly, Kana et al. showed that YAP could also induce Piezo1 expression and promote squamous cell carcinoma cell proliferation, suggesting the possibility of a positive feedback loop between Piezo1 and the YAP signaling cascade [40].

Using bioinformatics analysis based on the Chinese Glioma Genome Atlas (CGGA) dataset and the Cancer Genome Atlas (TCGA) network, Zhou et al. showed that Piezo1 is positively correlated with phosphatidylinositol-3-OH kinase (PI3K) and AKT2, which play important roles in regulating various cellular functions, such as growth, proliferation, and protein synthesis [33]. Piezo1 promotes proliferation by activating the PI3K/AKT/mTOR pathway. Downregulation of *Piezo1* suppresses Ca^2+^ influx, leading to the decrease of PI3K expression as well as AKT and mTOR phosphorylation [53]. This, in turn, reduces the activities of cyclin-dependent kinase 4 (CDK4) and Cyclin D1, and ultimately induces G0/G1 cell cycle arrest [43]. However, in HCC, Piezo1 did not affect the expression of AKT and phosphorylated AKT; instead, it promoted tumor progression by recruiting the ras-related protein Rab-5C (Rab5c) while activating TGF-β signaling, thus showing the possibility of different downstream target genes of Piezo1 in different cell types [37]. Together, these studies indicate that Piezo1 plays an important role in regulating the activation of proliferative signals that lead to excessive tumor cell proliferation (Table 2). However, as mechanistic studies are currently limited, further studies are needed to elucidate the molecular mechanisms underlying the effect of Piezo1 in promoting tumor cells proliferation potential.

### 3.2. Evading Growth Suppressors

Retinoblastoma protein (Rb) is a tumor suppressor that represses the transcription of genes required for the transition from the G1 to S phase, such as *Cyclin D*, *Cyclin E*, and *CDK2* [72]. Rb binds directly to the transactivation domain of E2F transcription factor 1 (E2F1), forming a complex that could, in turn, regulate the promoter activity of those genes [73]. Rb phosphorylation by Cyclin D1, CDK4, and CDK6 in the G1 phase releases its binding with E2F1, promoting the transcription of *Cyclin A*, *CDK1*, and *CDK2*, which are necessary for the S phase. Consequently, this negative regulation accelerates cell cycle progression [74].

Increased Rb phosphorylation has been found in various tumors, including cervical cancer, gastric cancer, and colorectal carcinoma, leading to a decrease in its inhibitory effect and subsequently promoting tumorigenesis [75,76,77,78]. Piezo1 is associated with this process and attenuates Rb tumor suppressive potential [31]. Piezo1 promotes Rb phosphorylation, resulting in the decrease of the expression of p21, a key regulator of cell cycle progression that inhibits various CDKs. This subsequently accelerates the cell cycle and tumorigenesis [31]. Furthermore, Piezo1 also could interact with p53, a well-known tumor suppressor which transcriptionally regulates the expression of *p21* as well as *B-cell lymphoma-2 (Bcl-2)-associated X* (*Bax*) [30,79,80]. The *p53* mutation could be found in more than 50% of tumor patients, and in patients with wild-type *p53*, its aberrant regulation is frequently found. Hence, increasing p53 protein accumulation has attracted attention as a potential antitumor therapeutic strategy. Given that Piezo1 suppression could increase p53 transcription level and reduce tumor growth [30], targeting Piezo1 might also become a potential antitumor therapeutic strategy. Collectively, these data suggest that Piezo1 suppresses the expression of growth inhibition factors and growth inhibitory signals in tumor cells (Table 2).

### 3.3. Apoptosis Resistance

Apoptosis is an autonomous programed cell death controlled by a series of genes, which involves a cascade of the activation of related factors, such as p53, Bcl-2 family, and caspase family [81]. Apoptosis is an important self-protection mechanism crucial for maintaining the integrity of the genomic information being passed to daughter cells, as it prevents cells with gene mutations and/or abnormal gene expression from proliferating [82]. The role of Piezo1 in regulating apoptosis remains intriguing. In some diseases, such as acute respiratory distress syndrome, hair shaft miniaturization, and osteoarthritis, Piezo1 exhibits a pro-apoptotic effect and contributes to the development of symptoms [83,84,85]. However, the effects and mechanisms of Piezo1 on tumor cell apoptosis are intricate.

Evading apoptosis, even in the presence of DNA damage and genomic instability, is an important hallmark of cancer [86,87]. Impaired p53 pathway is one of the most common mechanisms for inhibiting apoptosis in tumors. Inactivation or loss of p53 promotes the transcription of apoptotic factors such as *Bax* and/or suppresses that of antiapoptotic factors, such as *Bcl-2*, thus blocking apoptosis [88]. Piezo1 has been reported to inhibit tumor cell apoptosis and contribute to survival by promoting MAPK-mediated YAP phosphorylation and activating the YAP signaling pathway, suppressing caspase-3-dependent apoptosis in HCC cells [52]. Gao et al. showed, using human esophageal cancer cell lines and a mouse xenograft model, that Piezo1 evades apoptosis through the p53/Bax pathway, which increases the levels of both caspase-3 and cleaved-caspase-3 [30]. However, different results have been reported in other cancers. Jiang et al. found that the expression levels of Bax, Bcl-2-associated agonist of cell death, caspase-3, and caspase-9, are significantly increased after Piezo1 is activated by tensile force, indicating that Piezo1 contributes to apoptosis in osteosarcoma cells [41]. Similar results were obtained by ultrasonic-activated Piezo1 in pancreatic cancer cells and inducing Piezo1 expression using Yoda1 in colon cancer, which elevated mitochondrial membrane potential and induced cell death [29,42]. Therefore, Piezo1 showed paradoxical roles in apoptosis regulation in different tumors and should be further studied.

### 3.4. Sustained Angiogenesis

Solid tumor cells grow in a severe microenvironment with hypoxia, high matrix stiffness, and a lack of nutrients [89]. To overcome the limitations of such a harsh environment on cell growth, tumor tissue constantly generates dense blood vessels [90]. Hypoxia-inducible factor 1 alpha (HIF-1α) is a transcription factor that acts as a master regulator in regulating hypoxic response [91]. Under normoxia, HIF-1α protein is hydroxylated using oxygen as the substrate, leading to its ubiquitination/proteasomal degradation and a remarkably short half-life [92,93,94]. Upon exposure to hypoxia, HIF-1α is stabilized due to the lack of oxygen as the substrate for its hydroxylation, resulting in the accumulation of its protein, which in turn regulates the transcription of more than 160 target genes, most of them related to angiogenesis, cell survival, cell proliferation, and cell migration [95,96]. HIF-1α binds to the promoters of several angiogenic factors, including vascular endothelial growth factor (VEGF), platelet-derived growth factor-B (PDGF-B), fibroblast growth factor 2 (FGF2), stromal cell-derived factor-1 (SDF-1), and hepatic growth factor (HGF), and triggers their transcription [97,98,99,100,101]. Among them, VEGF initiates angiogenesis by promoting the formation of tube-like structures [102]. VEGF-A promotes endothelial cell permeability, as well as their proliferation and migration potentials, promoting the degradation of collagen, vascular basement membrane, and extracellular matrix (ECM) degradation by enhancing matrix metalloproteinases (MMPs) expression [103].

Piezo1 can induce tumor angiogenesis by sensing mechanical forces and transmitting downstream signals. Recent studies have shown that Piezo1 can induce tumor angiogenesis in an HIF-1α-dependent manner. Piezo1, HIF-1α, and VEGF are highly expressed in tumor tissues [30]. Piezo1 promotes HIF-1α expression at its transcriptional level through Ca^2+^ influx, as HIF-1α is a Ca^2+^-sensitive factor [104]. Furthermore, it can regulate HIF-1α at post-translational level by directly binding to HIF-1α, thereby suppressing HIF-1α hydroxylation and, subsequently, its ubiquitination/proteasomal degradation. This, in turn, stabilizes HIF-1α protein, activates the transcription of *VEGF*, and ultimately accelerates tumor angiogenesis and progression [54]. Additionally, Piezo1 could facilitate sprouting angiogenesis by functioning as an important mechanical force transducer. Vascular wall shear stress triggers Piezo1-mediated Ca^2+^ influx, promoting the expression levels of MMP2 and MMP1, which in turn promote sprouting angiogenesis [105]. Taken together, Piezo1 can respond to various mechanical forces to facilitate tumor angiogenesis by activating the HIF-1α pathway and promoting MMPs expression (Table 2).

### 3.5. Metastasis

Local invasion and distant metastasis are characteristics of malignant transformation. The complex process, in which tumor cells are subjected to various mechanical courses, such as compressive force, shear stress, and portal vein pressure, requires tumor cells to acquire a migration–invasion phenotype. This phenotype enables tumor cells to invade the basement membrane and propagate through the blood or lymph vessels [106]. Indeed, studies have shown that Piezo1 is generally upregulated in tumors and is closely linked with a poor prognosis, metastasis, and low survival rate in patients (Table 1) [34,107]. Inhibiting Piezo1 blocked the mechanically sensitive ion channel and resulted in the decrease of the migration potential of breast cancer cells, underscoring a possible role of Piezo1 in invasion and metastasis [24]. Furthermore, Piezo1 promotes colorectal carcinoma metastasis by increasing mitochondrial calcium uniporter (MCU) transcription that elevates mitochondrial membrane potential, activating the HIF-1α/VEGF pathway [29]. Piezo1 also promotes tumor cells’ invasiveness through a reciprocal regulation with matrix stiffness. Piezo1 localizes at the focal adhesions and activates integrin-focal adhesion kinase (FAK) signaling, leading to an increase in matrix stiffness; meanwhile, an enhanced mechanical microenvironment increases Piezo1 expression, thereby promoting glioma invasiveness [35].

Epithelial-mesenchymal transition (EMT) is a critical step in initiating the metastasis of transformed cells, in which epithelial cells lose their epithelial phenotypes, such as cell polarity and basement membrane detachment, while acquiring the characteristics of mesenchymal cells, including high migration and invasion, anti-apoptosis, and ECM degradation potentials. Recent studies showed that Piezo1 suppresses E-cadherin expression while promoting those of snail, E-cadherin, N-cadherin, and vimentin, which are typical markers of EMT, suggesting the role of Piezo1 in regulating EMT [27,37]. Hippo pathway is an evolutionarily conserved transduction pathway that controls organ size, and its deregulation promotes tumorigenesis [108,109]. Hippo pathway comprises upstream kinases, including mammalian Ste20-like serine/threonine kinases 1/2 (MST1/2), large tumor suppressor 1/2 (LATS1/2), and transcriptional co-activator YAP. Veglia et al. found that Piezo1 induces EMT in cholangiocarcinoma by reducing LATS1 phosphorylation, thereby inhibiting Hippo/YAP pathway and, subsequently, promotes YAP transcription [28].

Furthermore, tumor cells secrete ECM remodeling enzymes, such as membrane type 1 MMP (MT1-MMP), MMP-1, and MMP-2. These enzymes promote the degradation of various proteins that compose ECM; thereby disrupting histological barrier for tumor cell invasion [110]. Using the CGGA dataset and TCGA database, Zhou et al. performed transcriptomic analysis and revealed that Piezo1 expression in clinical glioma samples was positively correlated with genes involved in tumor angiogenesis, ECM organization, and metastasis [33]. Piezo1 could increase the activities of MMPs, including MMP-1, MMP2, MMP-3, MMP9, and membrane-bound MT1-MMP [25]. Meanwhile, extrusion pressure enhances the aggressiveness of breast cancer cells by increasing Ca^2+^ influx mediated by Piezo1 and activating downstream Src signaling transduction. This leads to increased MMPs expression and, subsequently, ECM degradation [56]. Piezo1 can also be coupled with calpain to cleave adhesion proteins and cytoskeletal matrix [52,111]. Furthermore, Piezo1 can also physically interact with trefoil factor 1 (TFF1) through its C-terminal to regulate integrin expression, which is crucial for regulating MMP2 and MMP9 activities [55]. Collectively, these results indicate that Piezo1 plays an important role in opening the initial invasion barrier of tumor cells that increases tissue invasion and metastasis.

Tumor cells migrate forward in the ECM by changing their morphology [112]. Piezo1 mediates the assembly of focal adhesion structures and cytoskeleton, which elevate cell contractile force and thus enhance the mobility of tumor cells [56,111]. In addition, it guides the formation of invadopodia to increase the invasive potential of tumor cells [113]. Ras homolog family member (Rho) GTPase family is one of the members of the ras superfamily [114]. Its family members, including ras-related C3 botulinum toxin substrate 1 (Rac1), ras homolog family member A (RhoA), and cell division cycle 42 (Cdc42), are engaged in the regulation of the morphology, cell-matrix adhesion, and cytoskeletal reorganization of tumor cells [115]. Zhang et al. found that Piezo1 inhibits the cumulative activation pattern of GTP-Rac1 and activates RhoA, leading to the well-organization of stress fibers F-actin [31].

However, the implications of Piezo1 in lung cancer contradict the other reported tumors, such as colon cancer, gastric cancer, and hepatocellular carcinoma [52]. Huang et al. showed that the *Piezo1* deletion variants in non-small cell lung cancer (NSCLC) lead to down-regulation of its expression, and, furthermore, the low level of Piezo1 is associated with poor prognosis in NSCLC patients [39]. Moreover, Piezo1 reduction promotes NSCLC cell migration in A549 cells and facilitates tumor growth in nude mice [38,39]. McHugh et al. indicated that the reduced Piezo1 in small cell lung cancer (SCLC) decreases calpain activity, thereby blocking the integrin-dependent cell migration. However, Piezo1 reduction also rearranges the cytoskeletal morphology into a ring like structure and increases the level of Tensin4, which is an amoeboid migration marker, suggesting that the downregulation of Piezo1 in SCLC promotes metastasis by increasing amoeboid migration [38]. However, whether Piezo1 reduction in other cancers causes amoeboid migration remains unknown, and the paradoxical functions of Piezo1 in lung cancer and other cancers remain to be further investigated. Taken together, while there are some conflicting results, the majority of studies have demonstrated that Piezo1 is beneficial for triggering tumor cell invasion and metastasis potentials by regulating their morphologies, promoting EMT, and clearing the invasion barrier (Table 2). Additional studies are needed to reveal the distinct role of Piezo1 in regulating these characteristics in different types of tumors.

### 3.6. Immune Evasion

In recent years, the regulatory functions of mechanical forces on immunity and inflammation have received increasing attention [116]. Despite the fact that the exact mechanism of mechanical force regulation on the immune system remains unclear, the role of Piezo1 in immune cells has been widely reported [117]. Piezo1 can modulate macrophage polarization, resulting in an increase in inflammation, which subsequently leads to delayed wound healing [118]. Furthermore, the mechanotransduction of Piezo1 is also crucial for the proinflammatory response of lung monocytes when exposed to cyclical pressure [119].

Tumors usually present in a state of immunosuppression and chronic inflammation, which are also essential hallmarks of cancer. Solid tumors contain many immune cells that recognize and destroy new tumor cells during cancer immunosurveillance [120,121]. However, these immune cells are regulated by tumor microenvironment, which could switch the immune response from the tumor-destructive mode to the tumor-promoting mode by secreting immunoregulation factor depending on its composition [122]. In a pan-cancer analysis, Piezo1 is remarkably associated with innate and adaptive immune responses, inflammation, as well as the infiltration of inflammatory cells, including lymphocytes, leukocytes, and neutrophils, suggesting that Piezo1 plays an important role in tumor immunity [123]. Myeloid-derived suppressor cells (MDSCs), which are pathologically activated neutrophils and monocytes, have strong immunosuppressive activity, and are considered closely related to tumor immune escape [124]. A recent study showed that Piezo1 increases MDSCs infiltration; furthermore, it inhibits intratumoral CD4^+^ memory T cells and CD8^+^ T cells accumulation, thereby promoting tumor progression [57]. Piezo1 could also upregulate the expression of histone deacetylase 2 (HDAC2), which suppresses Rb1 via epigenetic silencing, thereby expanding MDSCs and conferring a tumor immunosuppressive microenvironment (Table 2) [57]. Clinical breast cancer samples with Piezo1 high expression had fewer immunogenic characteristics, including the reduction of the activated CD^4+^ memory T cells and CD^8+^ T cells, which was associated with poor prognosis in breast cancer patients [27]. These studies revealed that Piezo1 induces inflammatory infiltration by increasing immunosuppressive cells and decreasing antitumor immune cells, thereby promoting the development of tumors. However, the evidence of Piezo1 in tumor immunization is still limited; thus, future studies are needed to explore the effects and mechanisms of Peizo1 on tumor immunity.

## 4. Conclusion and Perspective

As a critical component of mechanical conduction, Piezo1 has been reported to control physiological and pathological processes, such as innate immunity, bone formation, and various cancers [119]. Piezo1 transduces mechanical damage signals that drive tumorigenesis. In turn, constantly changing mechanical forces during tumor progression can further affect the outcome of the disease by altering Piezo1 expression. Piezo1 is highly expressed in most tumors and positively correlated with a poor prognosis (Table 1). Importantly, Piezo1 is closely related to cancer hallmarks [44]. Together, Piezo1 is a potential biomarker and predictor for tumors; furthermore, it is a potential antitumor therapeutic target.

However, the lack of specific inhibitors renders the clinical application of targeting Piezo1 [125]. While ruthenium red, amyloid β, GsMTx4, and margaric acid, as well as various polyunsaturated fatty acids, including eicosapentaenoic acid, docosahexaenoic acid, and arachidonic acid, have been identified recently as Piezo1 channel antagonists, they do not directly inhibit Piezo1 expression and/or activity; thus, their inhibitory effects are not specific [125,126]. For example, eicosapentaenoic acid decreases plasma membrane rigidity and bending stiffness that reduce the inactivation time constant of Piezo1, thereby damaging Piezo1 function [126]. Meanwhile, ruthenium red not only inhibits Piezo1, but also could inhibit a series of transient receptor potential vanilloid (TRPV), causing serious toxicity [63]. Furthermore, the molecular mechanisms of these inhibitors have not been fully elucidated [125]. Thus, while targeting Piezo1 is a potential antitumor therapeutic strategy, efforts are needed to further elucidate the detail molecular mechanisms regarding Piezo1 regulation and its regulation on downstream targets, as well as to develop specific, direct Piezo1 antagonists. Meanwhile, since the Piezo1-mediated Ca^2+^ signals in tumor cells extensively affect its downstream pathways, developing new drugs targeting its major downstream pathways is also a potential alternative approach. Moreover, Yoda1 and Jedi2, which are highly specific activators of Piezo1, can induce Piezo1 overexpression that causes the downstream factors alteration, thereby contributing to the study of its downstream signaling pathways [125,127,128].

Together, Piezo1 not only links up the physical changes surrounding the biological behaviors of tumor cells, but also has potential as a biomarker and antitumor therapeutic target.

## Figures and Tables

**Table 1:** Piezo1 expression in various cancers.

Cancer Types	Piezo1 Expression	Prognosis	Ref.
Breast cancer	Upregulated	Poor	[24,25,26]
	Upregulated	n/a	[27]
Cholangiocarcinoma	Upregulated	Poor	[28]
Colon cancer	Upregulated	Poor	[29]
Esophageal	Upregulated	n/a	[30]
Gastric cancer	Upregulated	Poor	[31,32]
Glioma	Upregulated	Poor	[33,34,35]
	Upregulated	n/a	[36]
Hepatocellular carcinoma	Upregulated	Poor	[37]
Lung cancer	Downregulated	Good	[38,39]
Oral squamous cell carcinoma	Upregulated	n/a	[40]
Osteosarcoma	Upregulated	n/a	[41]
Pancreatic cancer	Upregulated	Poor	[42]
Prostate cancer	Upregulated	n/a	[43]

Abbreviations: n/a: not available.

**Table 2:** Piezo1 regulation on hallmarks of cancer.

Hallmarks of Cancer	Phenotypes	Mechanisms	Ref.
Sustained proliferative signaling	Increases proliferation	MAPKs/YAP pathway activation	[52]
	Increases proliferation	YAP/Piezo1 pathway activation	[40]
	Increases proliferation	AKT/mTOR pathway activation	[53]
	Increases proliferation	Rab5c recruitment and TGF-β signaling activation	[37]
	Accelerates cell cycle	CDK4 and Cyclin D1 expression	[43]
Evading growth suppressors	Accelerates cell cycle	p21/Rb pathway suppression	[31]
	Inhibits antigrowth signals	p53/Bax expression suppression	[30]
	Inhibits antigrowth signals	p21/Rb expression suppression	[31]
Apoptosis resistance	Suppress apoptosis	Bax/Caspase-3 pathway suppression	[52]
	Suppress apoptosis	p53/Bax pathway suppression	[30]
Sustained angiogenesis	Promotes angiogenesis	HIF-1 stabilization	[54]
Metastasis	Induces EMT	Hippo/YAP pathway activation	[28,37]
	Promotes cell motility	RhoA/Rac1 pathway activation	[31]
	Promotes invasion	MCU/HIF-1/VEGF pathway activation	[29]
	Promotes invasion	TFF1/integrin pathway activation	[55]
	Induces ECM degradation	Src/MMPs pathway activation	[56]
	Promotes invasion	Integrin/FAK pathway activation	[35]
Immune evasion	Promotes tumor immunosuppression	MDSCs infiltration	[57]

Abbreviations: AKT: protein kinase B; Bax: B-cell lymphoma-2-associated X; Cdk4: cyclin-dependent kinase 4; FAK: focal adhesion kinase; HIF-1α: hypoxia-inducible factor 1 alpha; MAPKs: p38 mitogen-activated protein kinases; MCU: mitochondrial calcium uniporter; MDSCs: myeloid-derived suppressor cells; MMPs: matrix metalloproteinases; mTOR: mechanistic target of rapamycin; Piezo1: piezo-type mechanosensitive ion channel component 1; Rab5c: ras-related protein Rab-5C; Rac1: ras-related C3 botulinum toxin substrate 1; Rb: retinoblastoma protein; RhoA: ras homolog family member A; Src: Src proto-oncogene; TGF-β: transforming growth factor-β; TFF1: trefoil factor 1; VEGF: vascular endothelial growth factor; YAP: yes-associated protein.

## Abbreviations

AKT: protein kinase B; Bax: Bcl-2-associated X; Bcl-2: B-cell lymphoma-2; Ca^2+^: calcium; Cdc42: cell division cycle 42; CDK: cyclin-dependent kinase; CED: C-terminal extracellular domain; CGGA: Chinese Glioma Genome Atlas; CTD: intracellular C-terminal domain; E2F1: E2F transcription factor 1; ECM: extracellular matrix; EGFR: epidermal growth factor receptor; EMT: epithelial-mesenchymal transition; FAK: focal adhesion kinase; FGF2: fibroblast growth factor 2; HCC: hepatocellular carcinoma; HDAC2: histone deacetylase 2; HGF: hepatic growth factor; HIF-1: hypoxia-inducible factor 1 alpha; IH: inner helix; LATS1/2: large tumor suppressor 1/2; MAPKs: p38 mitogen-activated protein kinases; MCU: mitochondrial calcium uniporter; MDSCs: myeloid-derived suppressor cells; MMPs: matrix metalloproteinases; MST1/2: threonine kinases 1/2; MT1-MMP: membrane type 1 MMP; mTOR: mechanistic target of rapamycin; NSCLC: non-small cell lung carcinoma; OH: outer helix; P: phosphorylated; PDGF-B: platelet-derived growth factor-B; PH: peripheral helix; PI3K: phosphatidylinositol-3-OH kinase; Piezo1: piezo-type mechanosensitive ion channel component 1; Rab5c: ras-related protein Rab-5C; Rac1: ras-related C3 botulinum toxin substrate 1; Rb: retinoblastoma protein; RhoA: ras homolog family member A; SCLC: small cell lung cancer; SDF-1: stromal cell-derived factor-1; Src: Src proto-oncogene; TCGA: Cancer Genome Atlas; TGF-β transforming growth factor-; TFF1: trefoil factor 1; TRPV: transient receptor potential vanilloid; VEGF: vascular endothelial growth factor; YAP: yes-associated protein.
